# Antiviral potential of human IFN-α subtypes against influenza A H3N2 infection in human lung explants reveals subtype-specific activities

**DOI:** 10.1080/22221751.2019.1698271

**Published:** 2019-12-11

**Authors:** Aline da Rocha Matos, Katharina Wunderlich, Sebastian Schloer, Klaus Schughart, Robert Geffers, Martine Seders, Marlous de Witt, Anmari Christersson, Rainer Wiewrodt, Karsten Wiebe, Peter Barth, Andreas Hocke, Stefan Hippenstiel, Katja Hönzke, Ulf Dittmer, Kathrin Sutter, Ursula Rescher, Svetlana Rodionycheva, Nicoletta Matera, Stephan Ludwig, Linda Brunotte

**Affiliations:** a Institute of Virology Muenster, Westfaelische Wilhelms-University, Muenster, Germany; b Respiratory Viruses and Measles Lab, Oswaldo Cruz Institute, Fiocruz, Rio de Janeiro, Brazil; c Institute of Medical Biochemistry, Westfaelische Wilhelms-University, Muenster, Germany; d Department of Infection Genetics, Helmholtz Centre for Infection Research, Braunschweig, Germany; e Genome analytics, Helmholtz Centre for Infection Research, Braunschweig, Germany; f Department of Medicine A, Hematology, Oncology and Respiratory Medicine, University Hospital Muenster, Muenster, Germany; g Department of Thoracic Surgery, University Hospital Muenster, Muenster, Germany; h Gerhard-Domagk-Institute of Pathology, Westfaelische Wilhelms-University, Muenster, Germany; i Charité – Universitätsmedizin Berlin, corporate member of Freie Universität Berlin, Humboldt-Universität zu Berlin, and Berlin Institute of Health, Department of Internal Medicine/Infectious Diseases and Respiratory Medicine, Berlin, Germany; j Institute for Virology, University Hospital Essen, University of Duisburg-Essen, Duisburg, Germany

**Keywords:** Human lung explant, influenza, antiviral, IFN-α subtype, ISG induction, MxA

## Abstract

Influenza is an acute respiratory infection causing high morbidity and mortality in annual outbreaks worldwide. Antiviral drugs are limited and pose the risk of resistance development, calling for new treatment options. IFN-α subtypes are immune-stimulatory cytokines with strong antiviral activities against IAV *in vitro* and *in vivo.* However, the clinical use of IFN-α2, the only licensed subtype of this multi-gene family, could not prevent or limit IAV infections in humans. However, the other subtypes were not investigated.

Therefore, this study evaluated the induction and antiviral potential of all human IFN-α subtypes during H3N2 IAV infection in human lung explants. We found that subtypes with weak antiviral activities were preferentially induced during IAV infection in human lungs. Intriguingly, non-induced subtypes α16, α5 and α4 suppressed viral replication up to 230-fold more efficiently than α2. Furthermore, our results demonstrate that subtypes with stronger antiviral activities induce higher expression of IAV-specific restriction factors and that MxA expression is a determinant of the subtype-specific antiviral activity towards H3N2 IAV. These results corroborate that IFN-α subtypes exhibit differential antiviral activities and emphasize that subtypes α16, α5 and α4 should be further investigated for the prevention and treatment of severe infections with seasonal H3N2 IAV.

## Introduction

Influenza A viruses (IAV) cause recurring, highly infectious respiratory disease with mild to severe symptoms. It affects up to 5–10% of adults and 20–30% of children worldwide [1] and reaches 290000–650000 deaths annually [2]. Currently circulating IAV strains include the 2009 pandemic H1N1 strain (H1N1pdm09) as well as H3N2 strains [3]. Prevention of IAV infection can be achieved by annual vaccination and is of particular importance to elderly, infants, pregnant women and immune-compromised individuals, which are at high risk to develop severe symptoms. However, sporadic low efficacy of vaccines, due to mismatches between the circulating viruses and vaccine strains, and increasing scepticism towards the risks and benefits of vaccination raise the need for therapeutic treatments [4–6]. Additionally, introduction of new IAV strains with pandemic potential from animal reservoirs into the human population, for which a vaccine could not be timely available, remains to be a threat.

Currently available drugs for the treatment of severe IAV infection are limited and pose a high risk for resistance development. This is dramatically exemplified by the widespread resistance of current IAVs to M2-inhibitors [7] and former seasonal H1N1 viruses to neuraminidase inhibitors (NAIs) [8].

New anti-influenza drugs, like the cell fusion inhibitor arbidol, the ribonucleosid analogue favipiravir and the endonuclease inhibitor baloxavir have been approved but are not yet available worldwide [9–11]. Therefore, additional options to prevent and limit the severity of IAV infections need to be discovered.

In contrast to drugs that directly target viral proteins, stimulation of intrinsic host antiviral response mechanisms provides an efficient strategy for antiviral treatment with a reduced risk of resistance development [12]. A good example is given by the successful clinical application of PEGylated interferon alpha 2 (IFN-α2) for the treatment of chronic virus infections, such as with hepatitis C virus (HCV) [13].

IFN-α is a cytokine family belonging to type I IFNs, which possesses strong antiviral and immune-modulatory properties. In humans, the *IFNA* gene family is composed of 12 different subtypes encoded by 14 genes, including one pseudogene and two genes that encode identical proteins [14]. Induction of *IFNA* genes occurs in response to the recognition of pathogen-associated molecular patterns (PAMPs) by cellular pattern recognition receptors (PRRs), such as Toll-like receptors (TLRs) [15,16] and retinoic acid-inducible gene I (RIG-I) [17]. Secreted type I IFNs bind to the IFN-α receptor (IFNAR) in an autocrine and paracrine manner, leading to activation of the JAK/STAT pathway. Phosphorylated STATs assemble with IFN regulatory factor 9 (IRF9), forming the IFN-stimulated gene factor 3 (ISGF3) complex that binds to IFN-stimulated response elements (ISREs) in gene promoters and lead to the expression of IFN stimulated genes (ISGs), which possess diverse antiviral properties [18]. Many viruses have developed efficient mechanisms to counteract the induction of IFNs to suppress the expression of restriction factors. However, by exogenous application of purified IFNs, these counter mechanisms can be circumvented, opening the door for therapeutic interventions.

Currently, only subtype IFN-α2 is used for antiviral therapies, while the therapeutic potential of the other subtypes has remained largely unknown. Intriguingly, recent studies demonstrated that human IFN-α subtypes α8 and α14 are more potent inhibitors of HIV infection than IFN-α2. In contrast, for HBV, it was shown that mouse IFN-α subtypes α4 and α5 display stronger antiviral activity than IFN-α2, in a mouse model [19]. These reports suggest that IFN-α subtypes possess non-redundant immune-stimulatory and antiviral properties.

In addition to HIV and hepatitis viruses, also IAV are highly susceptible to exogenous IFN-α application *in vitro* and *in vivo* [20–23], suggesting high therapeutic potential against IAV infections. However, the translation of these findings to humans has, so far, been difficult and clinical studies were of limited success, either due to the lack of preventive or therapeutic effects or to the occurrence of adverse effects [24–28]. Intriguingly, most of these studies were based on the use of IFN-α2. Until today, only a limited number of reports assessed whether IFN-α subtypes differ in the antiviral activities against IAV *in vitro* or *in vivo,* with variable outcomes [29–31]. Consequently, the therapeutic potential of human IFN-α subtypes against IAV has not been investigated in a primary human study model to date.

To address this knowledge gap, the aim of this study was to determine the antiviral activities of all human IFN-α subtypes against a relevant seasonal IAV strain in human lung tissue. Our results demonstrated that IFN-α subtypes display individual antiviral properties against H3N2 IAV in human lung tissue. Most importantly, subtypes α16, α5 and α4 exhibited up to 230-fold higher antiviral activity compared to IFN-α2, but were not upregulated in human lung tissue upon *ex vivo* infection. Our results suggest, that human IFN-α subtypes α16, α5 and α4 should be further investigated for IAV treatments.

## Materials and methods

### Cells and viruses

Human alveolar epithelial cells (A549) and Madin-Darby canine kidney type II cells (MDCK) were cultivated in Dulbecco’s modified Eagle’s Medium (DMEM) (Sigma, Germany) supplemented with 10% fetal bovine serum (FBS) (Merck, Germany) and 1% Penicillin/Streptomycin (P/S) (Merck, Germany) at 37°C and 5% CO_2_. The IAV strain A/Panama/2007/1999 (H3N2) represents a prototypic seasonal H3N2 strain and is part of the virus collection of the German National Reference Centre for Surveillance and Nosocomial Infections (NRZ) of the Robert-Koch Institute. It was isolated in 1999 and was recommended as a vaccine strain by the WHO. The recombinant virus of this isolate was kindly provided by Thorsten Wolff (Robert-Koch Institute, Germany). Viruses were propagated on MDCKs for 72 h and viral titres were determined by standard plaque assay.

### MTT-Assay

The viability of A549 cells treated with IFN-α subtypes was assessed by using colorimetric MTT [3-(4,5-dimethylthiazol-2-yl)-2,5-diphenyltetrazolium bromide] assay (Sigma, Germany). 4.5 × 10^4^ A549 cells were incubated with 500 U/mL of the IFN-α subtypes for 48 h. MTT was added to the cells (5 mg/mL) for 4 h and plates were measured at 562 nm in a microplate reader (BioTeK, USA). Cells treated with 2 µM of the apoptosis-inducing kinase inhibitor staurosporine were used as positive control.

### Human lung explants

Tumor-free human lung explants were obtained from patients undergoing lung surgery at the University Hospital Muenster on the day of surgery. All patients gave their written consent to donate lung tissue for scientific purpose. Ethical approval was given by the ethical council of the Deutsche Ärztekammer (AZ: 2016-265-f-S). Lung tissue was recovered in Roswell Park Memorial Institute 1640 (RPMI) medium (Sigma, Germany) and stored at 4°C. The tissue was processed into tissue blocks of approximately 100 mg, placed in a 12-well plate and incubated overnight at 37°C with 5% CO_2_. For all experiments, untreated tissue of the same donor served as a negative control.

### Virus infection of human lung tissue

Virus was added to the medium of human lung tissue in the presence or absence of IFNs or BafA in RPMI+++ medium [RPMI supplemented with 2 mM L-Glutamin (Sigma, Germany), 1% P/S and 0.1% BSA]. 200 µL of the virus-containing medium were injected into the tissue using a syringe. After 1 h, tissues were washed to remove excess virus, placed in fresh RPMI+++ without IFNs or BafA and incubated at 37°C with 5% CO_2_ for 48 h. Supernatants were collected at 1, 24 and 48 h post infection (hpi) for titre determination.

### Treatment of lung tissue with IFN-α or Bafilomycin A

1000 U/mL of IFN-α subtypes or 250 nm of Bafilomycin (BafA) were added to the supernatants of lung explants. 200 µL of the drug-containing medium were injected into the tissue, followed by incubation for a total of 8 h (IFN-α) or 1 h (BafA) at 37°C and 5% CO_2_. For RNA extraction, IFN-treated tissue was transferred to 1 mL RNAlater (Sigma, Germany) 8 h (or 7 + 1 h with viral infection) post treatment (hpt). To assess the antiviral effect of IFN-α subtypes, virus infection (1 × 10^5^ PFU/mL) was performed 7 hpt, by adding virus to the drug containing supernatant for 1 h as described. For post-infection treatments, 2000 U/mL of the IFN-α subtypes were added to the supernatants 1 hpi.

### Isolation of total RNA from human lung tissue and qRT-PCR analysis

For total RNA isolation, the RNeasy Plus mini kit (Qiagen, Germany) was used, according to manufacturer`s instructions. Tissue blocks were transferred to Lysing Matrix A tubes (MP Biomedicals, Germany) in RLT buffer supplemented with 1% β-Mercapthoethanol (Sigma, Germany). Tissue was lysed using FastPrep®-24 (MP Biomedicals, Germany) by applying six rounds of lysis at default settings (4 m/s, 20 s). Homogenates were centrifuged and RNA was purified from the supernatant. Total RNA was reverse transcribed with oligo (dT) primers and RevertAid H Minus Reverse Transcriptase (Thermo Fisher Scientific, USA). qRT-PCR was carried out in duplicates using a LightCycler 480 II (Roche, Germany) by using specific primer sequences (Supplementary Table 1). Commercial primers were used for analysis of *IFITM3* and *IFN-β* mRNA (Qiagen, Germany). mRNA expression data were normalized to glyceraldehyde 3-phosphate dehydrogenase (*GAPDH*) and analysed by using the 2^−ΔΔCT^ method.

### RNA sequencing and data analysis

RNA integrity was controlled on Agilent Technologies 2100 Bioanalyzer (Agilent Technologies, Germany). The RNA sequencing library was generated from 500 ng total RNA using Dynabeads mRNA DIRECT Micro Purification Kit (Thermo Fisher Scientific, USA), for mRNA purification, followed by NEBNext Ultra II Directional RNA Library Prep Kit (New England BioLabs, USA), according to manufacturer’s protocols. The libraries were sequenced on Illumina NovaSeq 6000 using NovaSeq 6000 S1 Reagent Kit (200 and 300 cycles, paired end runs) with an average of 7.9 × 10^7^ reads per sample.

Reads were quality checked with package FastQC (version 0.11.4, reference see link below), then trimmed using Trimgalore (version 0.4.4, reference see link below) with default settings. Trimmed reads were mapped to human genome annotation hg38 (ENSMBL hg38 release 91) and H3N2 virus (gb:CY034100|A/Panama/2007/1999) using STAR (version 2.5.2b [32]) with default settings. Mapped reads were counted using RsubRead (version 1.32.4) [33]. For the RNA sequencing data, analysis and visualization of expression data was performed using the R software package (version 3.4.0) [34]. Raw counts from human genome and virus mapping were combined and normalized using DESeq2 (version 1.16.1) [35]. For identification of differentially expressed genes (DEGs), the LIMMA package (version 3.32.4) [36] using Benjamini-Hochberg (BH) correction for multiple testing [37] was used. DEGs were identified at the level of the probe sets by using a threshold of more than a 1.5 fold (log2 of 0.5849625) difference in expression levels and an adjusted *p*-value of < 0.1. Sequencing results are accessible at Gene Expression Omnibus using dataset identifier GSE135069: https://www.ncbi.nlm.nih.gov/geo/query/acc.cgi?acc=GSE135069.

### Lung tissue viability assay

Human lung explants were cultured for 48 h. Supernatants were analysed for lactate dehydrogenase (LDH) release using the Cytotoxicity LDH Detection Kit (Clontech, USA). As a positive control, lung tissue was disrupted by using FastPrep®-24 (MP Biomedicals, Germany), as described. Optical density (OD) was measured at 450 nm using a MicroLumat Plus LB96 V (Berthold Technologies, Germany). Values were normalized to the individual weights of the tissue explants. Cytotoxic effects of IFN-α subtypes were assessed by treating human lung tissue with 2000 U/mL of each subtype for 48 h. Detection of LDH in the supernatants was performed at 24 and 48 hpt.

### Analysis of secreted cytokines from human lung tissue

Supernatants from infected human lungs were analysed at 24 hpi using the LEGENDplex™ Human Anti-Virus Response Panel (BioLegend, USA). Cytokine capturing was performed according to the manufacturer’s protocol in filter plates. Bead-bound cytokines were measured on a FACSCalibur Cytometer (Becton Dickinson). Concentrations were calculated using the LEGENDplex™ Data Analysis Software (BioLegend, USA). Amounts of secreted IL6 and IL8 were evaluated at higher dilution using a customized two-plex panel (LEGENDplex).

### Recombinant IFN-α subtypes

All 12 human IFN-α subtypes were expressed in *Escherichia coli*. Refolded proteins were purified by anion-exchange and size-exclusion chromatography. Endotoxin levels were determined using the ToxinSensor Kit (Genescript, USA) and were below 0.0025 endotoxin units (EU/mL). For quantification of the IFN-α units, subtypes were titrated on human retinal pigment epithelial cells stable transfected with a reporter plasmid containing 5 copies of a consensus ISRE element in front of a firefly luciferase gene (Stratagene, USA) [38,39].

### Immunohistochemistry

Detection of the viral nucleoprotein (NP) in infected lung explants was performed by immunohistochemistry. Lung explants were fixed in 4% paraformaldehyde (PFA) at 48 hpi. Following dehydration in ascending 2-propanol dilutions, tissues were embedded in paraffin and cut into 4 µm sections. Antigens were demasked in 1x Lab Vision™ PT Module™ solution (Thermo Fisher Scientific, USA) at 95°C for 20 min. Tissue sections were blocked in 10% FBS with 1% Triton-X100 for 30 min. Viral NP was stained using anti-NP antibody (G501, provided as a gift by Robert Webster, St. Judes Childrens Hospital, Memphis, USA) and a species-specific secondary antibody. Antigen visualization was performed using the Vectastain ABC-AP Kit (Vector Laboratories, USA). Cell nuclei were counterstained using Mayer’s hemalum solution (Carl Roth, Germany).

### MxA knockout cells

Lentiviral particles harbouring a single guide (sg) RNA targeting the human *MX1* gene or a control sgRNA [40] were kindly provided by Georg Kochs (University Freiburg, Germany). Lentiviral stocks were used for transduction of A549 cells to generate MxA knockout (KO) (ΔMxA) and control (Ctr) cells. MxA KO was validated by western blot analysis using primary antibodies against MxA (kindly provided by Georg Kochs). Successfully transduced cells were selected with 4 µg/mL of puromycin (Sigma, Germany).

5 × 10^5^ A549 ΔMxA and Ctr cells were either treated with IFN-α subtypes (500 U/mL) or mock-treated for 7 h. This was followed by infection (MOI 0.01) by adding the virus to the supernatant for an additional 1 h, resulting in 8 h total drug treatment. Virus-containing supernatant was replaced with fresh medium and supernatants were collected 12, 24 and 48 hpi and titrated on MDCK cells.

### IC_50_ determination

A549 cells were pre-treated with different concentrations of each subtype (10^−2^10^−5^ U/mL), for 7 h, followed by infection (MOI 0.01), for 1 h, as described. Supernatants were collected 48 hpi and titrated on MDCK cells. The IFN-α subtype concentration required to decrease the viral titres by 50% (IC_50_) was calculated using GraphPad Prism.

### Statistical analysis

Statistical analysis was performed with GraphPad Prism (GraphPad Software Inc., USA) unless otherwise specified. mRNA expression, by qRT-PCR, and secreted cytokine levels, by multiplex panels, in mock-infected and infected lungs was compared by paired Student's T-test. Experiments with repeated measurements (RM) were compared by two-way or three-way ANOVA with the indicated post-tests for multiple comparisons. IC_50_ values were determined by non-linear regression. Correlation analysis between IFN-α subtypes antiviral activity and ISGs induction was performed by linear regression analysis to calculate R^2^ and *p*-values.

## Results

### Replication and host response to H3N2 IAV infection in human lung tissue

**Figure 1.**
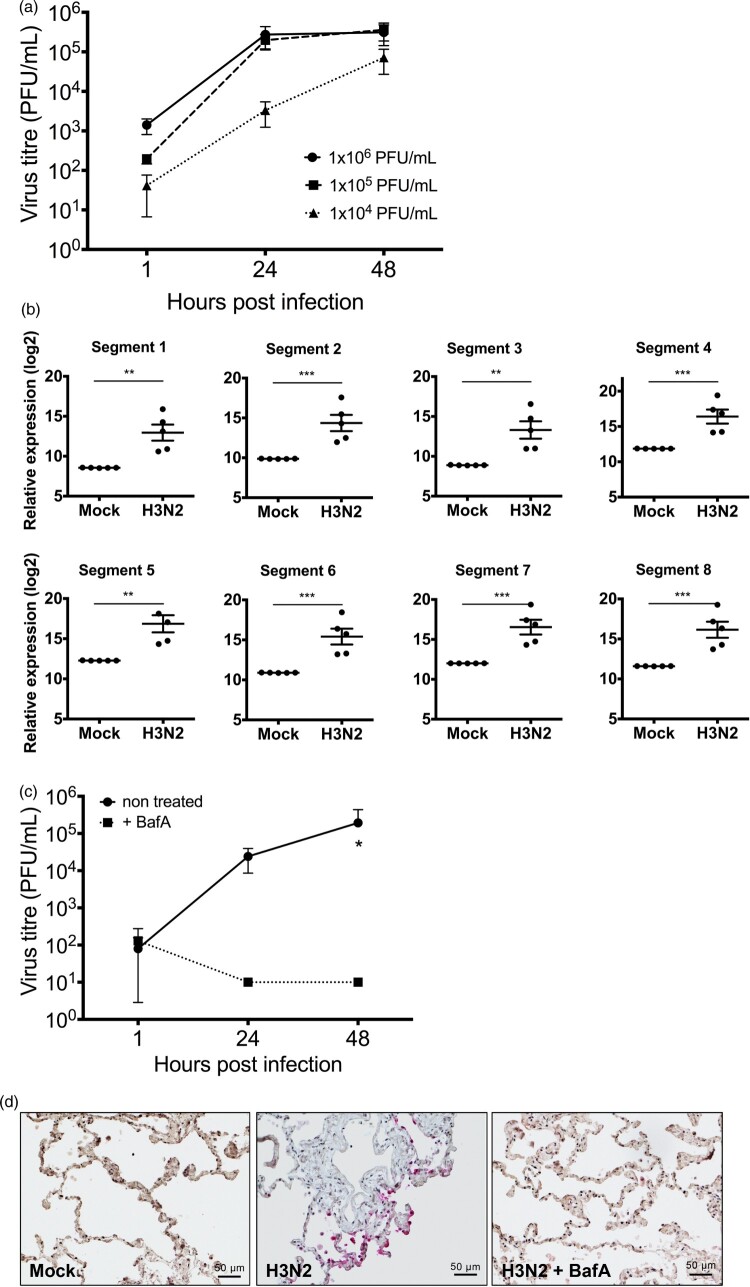
Replication of H3N2 IAV in human lung explants. (a) Lung tissue was infected with the indicated amounts of H3N2 IAV particles. Viral titres were determined by plaque assay. *n* = at least 3 independent donors. (b) RNA-sequencing of total RNA isolated from human lung tissue 24 hpi. *n* = 5 independent donors. Bars represent mean (± SEM). ** *p* ≤ 0.01 *** *p* < 0.001; Multiple testing with BH correction. (c) Treatment of human lung tissue with bafilomycin A (BafA) inhibits H3N2 replication. Human lung tissue was pre-incubated with 250 nM BafA for 1 h prior to infection with H3N2. Each time point represents mean (± SEM). Two-way ANOVA, Sidak’s multiple comparison test. *p ≤ 0.05 (d) Immunohistochemistry of infected and non-infected lung explants. Viral nucleoprotein (NP) and cell nuclei were stained using anti-NP antibody (pink staining) and Mayer’s hemalum solution (blue staining), respectively.

Previous to the assessment of the antiviral properties of IFN-α subtypes against seasonal H3N2 IAV, viral replication kinetics and the host immune response in human lung tissue were analysed. *Ex vivo* infection of human lung tissue demonstrated stable viral replication over four log_10_ steps within 48 h (Figure 1(A)) and significant accumulation of viral RNAs derived from all eight viral genome segments as determined at 24 hpi (Figure 1(B)). Viability of the explants was verified by measuring the release of LDH in non-infected tissues for up to 48 h (supplementary Figure 1). These results demonstrate that human lung explants support IAV replication and reinforce that lung explants provide a suitable model to study pathogen host interactions of respiratory viruses in primary human lung tissue [41]. To assess whether this model can also be applied to investigate drug treatments against IAV, we used the macrolide antibiotic bafilomycin A (BafA) to inhibit H3N2 IAV replication in lung tissue. BafA is an inhibitor of vacuolar-type H^+^-pumps and prevents endosomal acidification. As expected, pre-treatment with BafA for 1 h abrogated viral replication, resulting in significantly reduced virus titres (Figure 1(C)) and the absence of NP positive cells in BafA-treated and infected lung tissue sections (Figure 1(D)).

**Figure 2.**
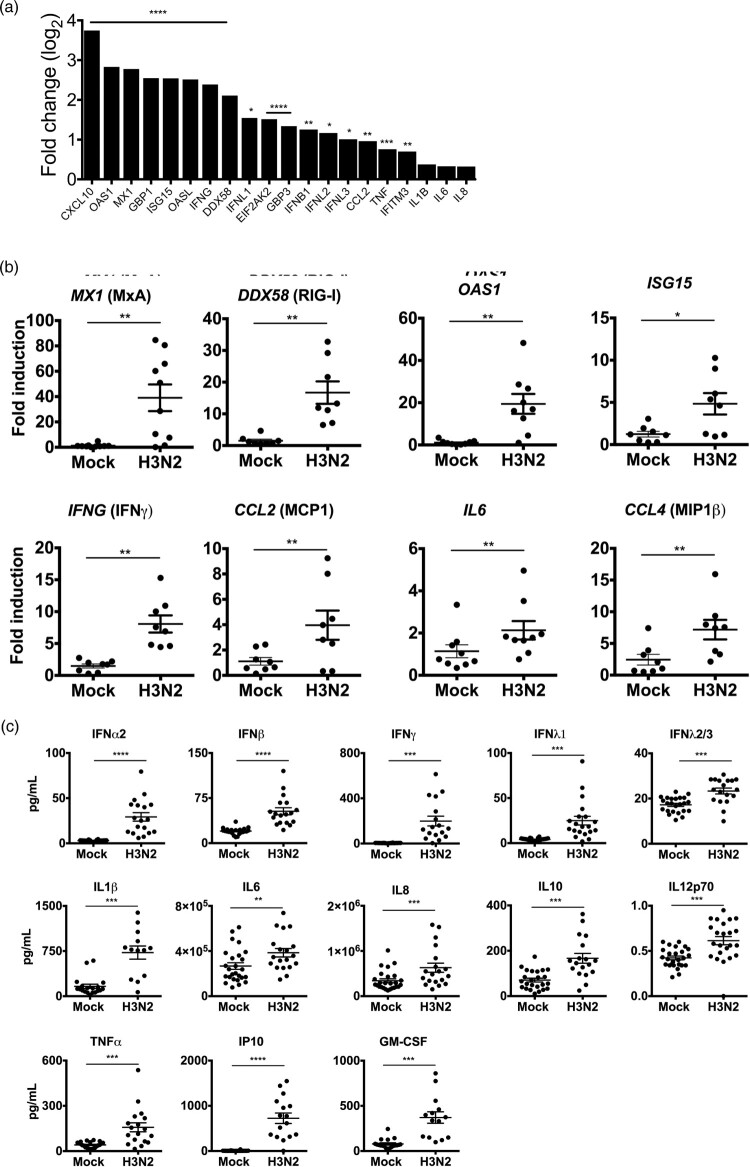
Innate immune response to H3N2 IAV infection in human lung explants. Tissue was infected with 1 × 10^5^ PFU/mL of H3N2 IAV or mock-infected. (A) Total RNA was isolated 24 hpi for RNA-sequencing. mRNA expression levels are depicted as mean log_2_ fold change compared to mock-infected tissue. *n* = 5 independent donors. Multiple testing with BH correction. (B) mRNAs of individual ISGs and cytokines were analysed by qRT-PCR. GAPDH was used as the housekeeping gene. (C) Secreted cytokines were analysed in the supernatants at 24 hpi. Individual results of infected and mock-infected lung tissues are derived from the same donor. Bars represent mean (±SEM). *p < 0.05 **p < 0.01 ***p < 0.001 ****p < 0.0001; paired T-test.

To characterize the host innate immune response to H3N2 IAV infection in human lung explants at 24 hpi, RNA sequencing analysis was performed. This revealed significant transcriptional upregulation of antiviral restriction factors including *OAS1, MX1* (MxA)*, GBP1, ISG15, OASL, DDX58* (RIG-I)*, EIF2AK2* (PKR)*, GBP3* and *IFITM3* and the pro-inflammatory cytokines and chemokines *CXCL10* (IP10)*, CCL2* (MCP1) and *TNF* (TNF-α) (Figure 2(A) and supplementary Table 2). To substantiate these findings, mRNA upregulation of *MX1, DDX58, OAS1* and *ISG15* and cytokines *IFNG, CCL2, IL6* and *CCL4* was verified by qRT-PCR (Figure 2(B)). Additionally, to evaluate the secretion levels of IFNs and other pro-inflammatory cytokines from infected human lung tissue, we performed a FACS-based multiplex analysis from the supernatants at 24 hpi (Figure 2(C)). This provided evidence that H3N2 IAV infection induced strong upregulation and secretion of type I IFNs (IFN-α2, IFN-β), type II (IFN-γ) and type III IFNs (IFN-λ1, λ2/3) and verified that the upregulation of pro-inflammatory cytokines also occurred at translational level. Induction of type I, II and III IFNs suggests the involvement of epithelial (type I and III IFNs) as well as immune cells (type II IFNs) in the antiviral response to H3N2 IAV infection in the human lung.

**Figure 3.**
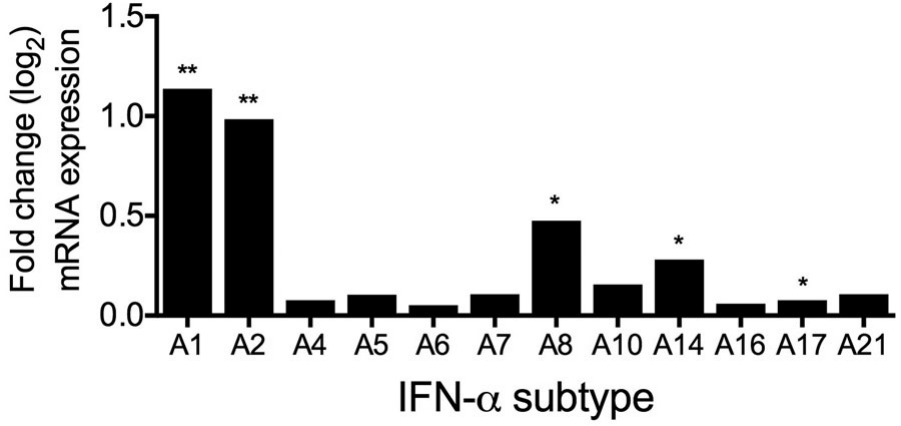
Induction of IFN-α subtypes mRNA levels by H3N2 IAV infection in human lung explants. Total RNA was isolated 24 hpi for RNA-sequencing. mRNA levels are depicted as mean log_2_ fold change, compared to mock-infected tissue from the same donor. *n* = 5 independent donors. *p < 0.05 **p < 0.01. Multiple testing with BH correction.

To characterize the early antiviral immune response in more detail, we were interested to determine whether other IFN-α subtypes than IFN-α2 were induced after infection. RNA sequencing results revealed that mRNAs of subtypes *IFNA1*, *A2*, *A8*, *A14* and *A17* were significantly upregulated during H3N2 IAV infection (Figure 3).

In conclusion, our results demonstrate that human lung explants induce a complex innate immune response following infection with H3N2 IAV, which is manifested by the expression of antiviral restriction factors, pro-inflammatory cytokines, type I, II and III IFNs and a unique pattern of IFN-α subtypes.

### IFN-α subtypes differentially suppress replication of H3N2 IAV in human lung tissue

Our results demonstrated that several IFN-α subtypes, including α1, α2, α8, α14 and α17 were upregulated in IAV infected lung tissue. It is known that the clinically approved subtype IFN-α2 displays antiviral activity against IAV, however, for the other subtypes, information on their antiviral properties against IAV is not available. Therefore, we were interested to analyse the antiviral activities of all human IFN-α subtypes against H3N2 IAV in our model.

Type I IFNs are reported to induce a rapid immune response accompanied by high expression levels of ISGs. In order to compare the antiviral activities of the subtypes, we exogenously applied 1000 U/mL of the specific subtypes to the supernatants of human lung explants for 7 h prior to the addition of 1 × 10^5^ PFU/mL of H3N2 IAV for 1 h. IFN and virus-containing supernatants were removed and viral replication was analysed by titrating virus titres in the supernatants 1, 24 and 48 hpi by plaque assay.

**Figure 4.**
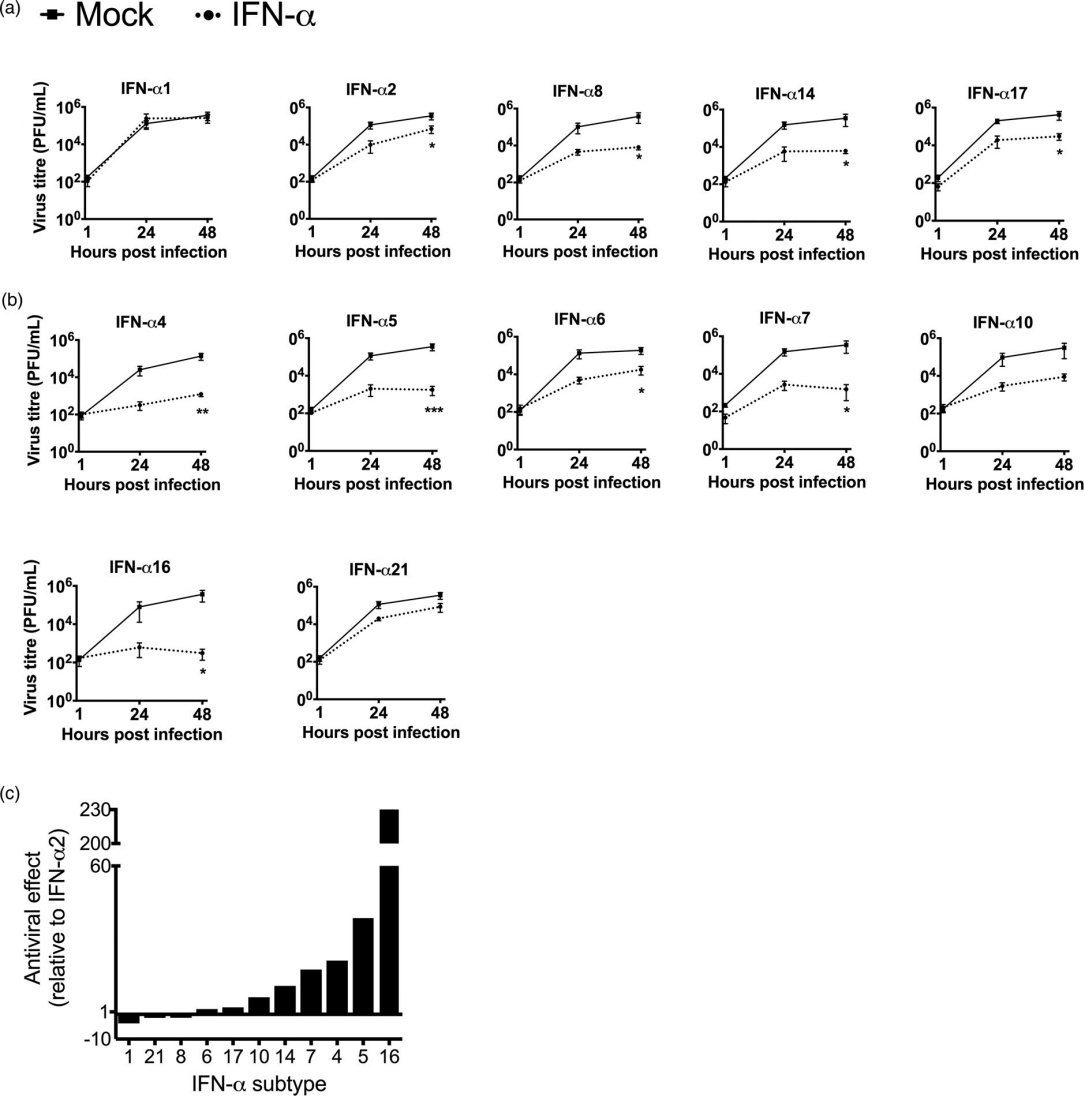
Inhibition of H3N2 IAV replication by IFN-α subtypes. Tissues were treated with human IFN-α subtypes (1000 U/mL) for 7 h before 1 × 10^5^ PFU/mL of H3N2 IAV were added to the medium with for an additional hour. (A) Antiviral activity of IFN-α subtypes significantly induced during infection. (B) Antiviral activity of the remaining IFN-α subtypes not induced during infection. Viral titres were determined by plaque assay. Time points represent mean (± SEM). (C) Antiviral effects of IFN-α subtypes against H3N2 were calculated over mock-treated tissue (n-fold reduction in viral titres) and compared to IFN-α2, at 48 hpi. *p < 0.05 **p < 0.01 ***p < 0.001; two-way ANOVA multiple comparison test.

We first analysed the subtypes that were expressed during infection and observed that, except for IFN-α1, all subtypes suppressed H3N2 IAV replication by 1–2 log_10_ step(s) at 48 hpi (Figure 4(A)). However, IFN-α2, which was used for the majority of studies involving IAV, displayed weaker antiviral activity than α14 and α17 (Figure 4(A and C)).

Next, we also investigated the antiviral potential of the seven remaining subtypes, which were not induced during H3N2 IAV infection. All of these subtypes (α4, α5, α6, α7, α10, α16 and α21) also exhibited antiviral activity, however, the degree of suppression strongly differed between the individual subtypes (Figure 4(B)). Direct comparison with the inhibitory effect of IFN-α2 revealed that subtypes α1, α21 and α8 are less antiviral, while subtypes α6, α17, α10, α14, α7, α4, α5 and α16 displayed higher antiviral activity against H3N2 IAV (Figure 4(C)). The three most potent subtypes α16, α5 and α4 reduced virus growth by 3–4 log_10_ steps at 48 hpi. To rule out that viral titres were reduced due to cytotoxic effects of the IFNs, cell viability of IFN-treated A549 cells was tested following 48 h incubation with 500 U/mL of the individual subtypes. However, only subtypes α2 and α5 significantly reduced cell viability to 94 and 88%, respectively, under these conditions (Supplementary figure 2).

**Figure 5.**
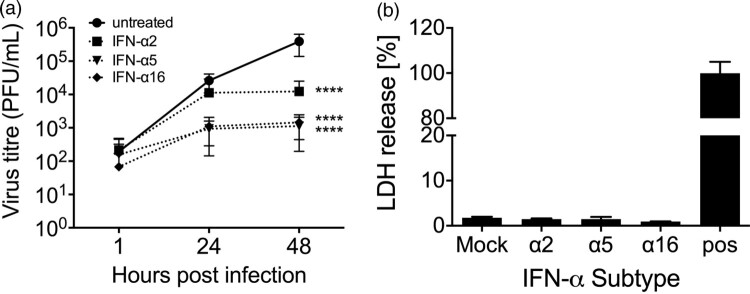
Antiviral activity of IFN-subtypes post-infection. (A) Human lung tissue was infected with 1 × 10^5^ PFU/mL of H3N2 IAV for 1 h followed by application of 2000 U/mL of the indicated IFN-α subtypes to the infection medium. Samples of the supernatants were collected at 1, 24 and 48 hpi and titrated on MDCK cells. Each time point represents mean (± SEM). *n* = 4 independent donors. Two-Way ANOVA, Sidak’s multiple comparison test. ****p < 0.0001. (B) Cytotoxicity of human IFN-α subtypes in human lung explants. Presence of LDH was measured in the supernatant of human lung explants treated with 2000 U/mL of the indicated IFN-α subtypes at 48 hpt. Mechanically disrupted lung tissue was used as a positive control (pos). All values were normalized to the weight of the individual explants.

We also tested whether subtypes α5 and α16 are more potent in restricting viral replication in a therapeutic setting, as compared to α2. Lung explants were infected and 1 hpi 2000 U/mL of the subtypes α2, α5 and α16 were added to the supernatants for 48 h. This demonstrated that α5 and α16 strongly reduced viral replication by more than 2 log_10_ steps while α2 reduced viral titres only by 1.5 log_10_ steps (Figure 5(A)). Cytotoxic effects due to the high IFN-α concentrations were excluded by measuring LDH release (Figure 5(B)). We suspected that viral titres could be reduced by the induction of an antiviral response in MDCK cells during plaque assay due to the high IFN-α concentrations in the supernatants. To assess this possibility, we treated lung tissue with the IFNs for 48 h and took samples at 1, 24 and 48 hpt. Subsequently, we spiked in 1 × 10^6^ virus particles to the supernatants and performed plaque assay to assess the induction of antiviral activity in the MDCK cells. However, as shown in supplementary figure 3, viral titres were not reduced.

**Figure 6.**
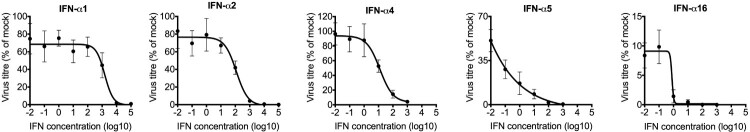
Dose response curves for human IFN-α subtypes against H3N2 IAV in A549 cells. Cells were treated for 8 h with different concentrations of each subtype (10^−2–^10^5^ U/mL) and then infected with H3N2 (MOI 0.01). Viral titres were determined by plaque assay at 48 hpi. Data points represent mean titre of at least 3 independent experiments.

To confirm the antiviral activities of the IFN-α subtypes in a second experimental model, we determined the IC_50_ of selected subtypes in A549 cells (Figure 6 and Supplementary Figure 4). We found that also in A549 cells α16 and α5 were the most potent subtypes (IC_50_ < 0.01 U/mL), while α1 and α21 (IC_50_ = 1519.0 and 209.8 U/mL, respectively) were the least potent (Table 1). Compared to IFN-α2 (IC_50_ = 116.7 U/mL), α16 and α5 were > 10000-fold more potent in inhibiting H3N2 IAV replication. Interestingly, in contrast to the results in human lung tissue, α14 (IC_50_ = 1.92 U/mL, supplementary figure 4) was more potent than α4 in A549 cells, which may indicate differences between the experimental models.

**Table 1.** IC_50_ values of human IFN-α subtypes against IAV H3N2.

IFN-α	IC_50_ (U/mL)	IC_50_ (pg/mL)	Antiviral activity Compared to IFN-α2 (n-fold)
1	1519.00	157095.1	−13.0
**2**	**116**. **70**	**1652**. **3**	**1**. **0**
4	12.40	29.5	9.4
5	<0.01	<0.9	>11670
14	1.92	15.1	60.8
16	<0.01	<2.1	>11670
21	209.80	427.7	−1.8

Note: At least 3 independent experiments were performed. Quantified values were analyzed by non-linear regression to determine IC_50_ values. The relative potencies were calculated by dividing the IFN-α2 IC_50_ by the IC_50_ of the other subtypes.

In summary, our data demonstrate that IFN-α subtypes comprise individual capacities to inhibit the replication of H3N2 IAV. In total, eight of 11 IFN-α subtypes displayed higher antiviral activity than IFN-α2, with α16, α5 and α4 being the most potent subtypes, suggesting that they are more suitable candidates for the therapeutic treatment of IAV infections.

### IFN-α subtype-dependent induction of IAV restriction factors in human lung tissue

**Figure 7.**
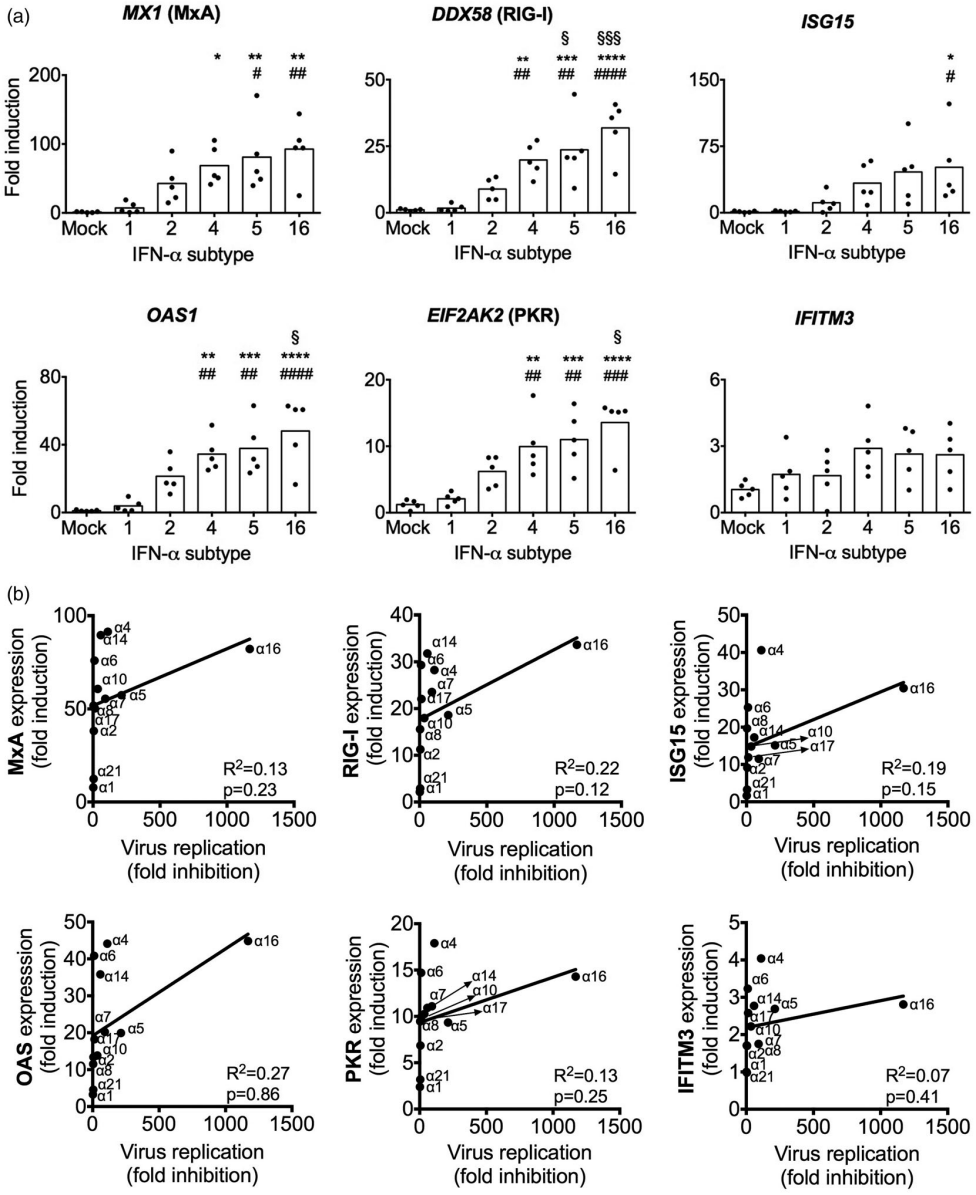
Induction of antiviral ISGs by IFN-α subtypes. (A) mRNA induction by the indicated subtypes (1000 U/mL) was analysed by qRT-PCR at 8 hpt. GAPDH was used as the housekeeping gene. *n* = 5 independent donors. Bars represent mean fold induction. *p < 0.05 **p < 0.01 ***p < 0.001 ****p < 0.0001 versus mock; #*p* < 0.05 ##*p* < 0.01 ###*p* < 0.001 ####*p* < 0.0001 versus α1; §*p* < 0.05 §§§*p* < 0.001 versus α2. (B) Correlation analysis of ISGs induction and virus fold inhibition of each subtype against mock-treated tissue was performed by linear regression. R^2^ and *p*-values were calculated.

To analyse whether the differences in the antiviral activities of the IFN-α subtypes are correlated to differences in the induction of antiviral restriction factors, we stimulated non-infected human lung explants for 8 h with selected subtypes, representing low (α1), moderate (α2) and high (α4, α5 and α16) antiviral activities. mRNA expression levels of the IAV restriction factors [42,43] *MX1* (MxA)*, DDX58* (RIG-I)*, ISG15, OAS1, EIF2AK2* (PKR) and *IFITM3* were determined (Figure 7(A)). In correlation with the lack of pronounced antiviral activity, α1 and α2 did not significantly upregulate any of these ISGs, compared to mock-treated lungs. In contrast, subtype α16, the most potent anti-H3N2 IAV subtype, significantly increased expression of all ISGs but *IFITM3*. Similarly, α5 and α4 significantly upregulated expression of *DDX58, OAS1* and *EIF2AK2,* compared to mock and α1, in addition to significantly inducing *MX1*, in comparison to mock. Of note, we did not detect significant upregulation of *IFITM3* mRNA levels by any tested subtypes. Overall, this supports the hypothesis that stronger antiviral activity correlates with increased expression of IAV-specific restriction factors.

To confirm this hypothesis and analyse whether the expression of a specific factor may be more important, we performed a correlation analysis of ISG induction and antiviral activity with all subtypes (Figure 7(B)). However, this analysis revealed very low correlation coefficients (R^2^) for all of the tested restriction factors, indicating that the subtype-specific antiviral activity against H3N2 is not determined by one of these ISGs alone.

### Suppression of IAV replication by IFN-α subtypes is differentially dependent on the restriction factor MxA

**Figure 8.**
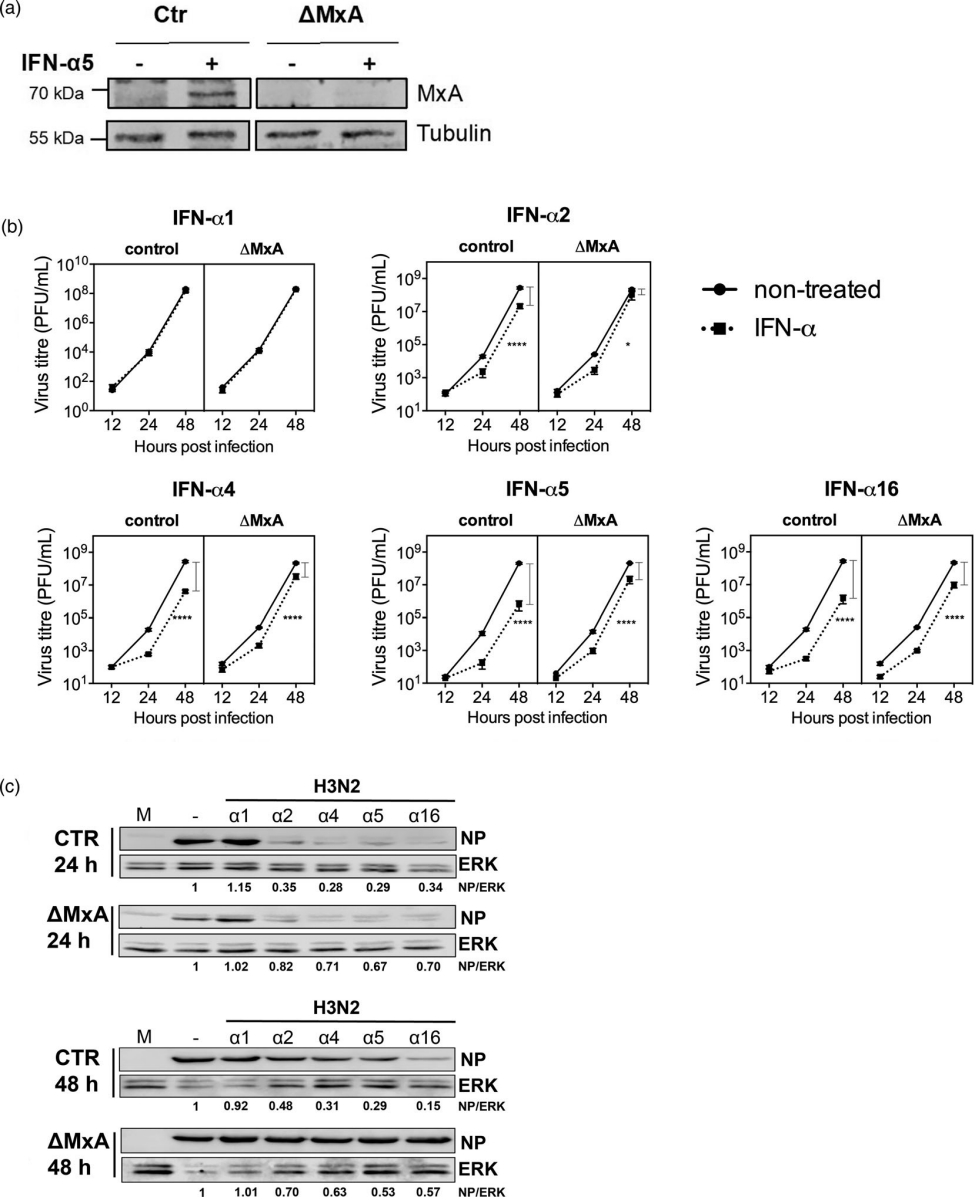
MxA-dependency of the antiviral activity of IFN-α subtypes against H3N2 IAV. (A) Western blot analysis of A549 control (Ctr) and MxA KO (ΔMxA) cells, treated (+) or mock-treated (-) with IFN-α5 (500 U/mL) for 8 h. (B) Ctr and ΔMxA A549 cells were treated with IFN-α subtypes (500 U/mL) for 8 h and infected with H3N2 IAV (MOI 0.01). Viral titres in the supernatants were determined by plaque assay. Grey bars indicate the difference in viral titres between non-treated and IFN-treated cells within the respective cell lines at 48 hpi. *n* = 4. Data are presented as mean (±SEM). Ctr mock versus Ctr IFN-α: **p ≤ 0.01; ***p ≤ 0.001. ΔMxA mock versus ΔMxA IFN-α: *p ≤ 0.05; ****p ≤ 0.0001. Three-way ANOVA, Tukey’s multiple comparison test. (C) Western blot analysis of viral NP expression. ERK was stained as a loading control. Cells were treated as in B and lysed at 24 and 48 hpi.

Due to the importance of MxA for the inhibition of IAV [21], we were interested to determine to which extent the subtype-specific antiviral activity was dependent on the presence of this restriction factor. For that matter, MxA KO A549 cells were generated using CRISPR-Cas9 technology (Figure 8(A)). We compared H3N2 IAV replication kinetics in Ctr and MxA KO cells, either mock-treated or pre-treated with IFN-α subtypes. We observed that the treatment with all tested IFN-α subtypes, except for α1, leads to a reduction in viral replication and NP expression in both Ctr and MxA KO cell lines, as compared to the non-treated cells. However, the reduction of viral titres and NP expression was less pronounced in IFN-treated MxA KO cell than in the Ctr cells (Figure 8(B and C)). Most importantly, absence of MxA differentially affected the antiviral activity of the tested subtypes as determined by the viral titres in IFN treated KO cells. In MxA KO cells, the antiviral activities of α4 and α16 were only reduced by 10.53 and 8.44-fold, respectively. In contrast, the antiviral activity of α5 was reduced by 33.58-fold, indicating that α5 depends to a greater extent on MxA for its antiviral activity against H3N2 IAV than α4 and α16 (Table 2).

**Table 2.** MxA-dependency of the antiviral activity of IFN-α subtypes against H3N2 IAV.

IFN-α subtype	Viral growth in presence of MxA(%)	Viral growth in absence of MxA (%)	Dependency on MxA (n-fold)
MOCK	100.0	100.0	1.00
1	73.48	87.91	1.20
2	8.12	48.18	5.93
4	1.51	15.91	10.53
5	0.31	10.43	33.58
16	0.55	4.61	8.44

Note: Viral growth in cells pre-treated with IFN-α subtypes was calculated as percentage of mock treated cells at 48 hpi in presence or absence of MxA. N-fold dependency on MxA was determined by dividing viral growth in MxA absence (%) by viral growth in MxA presence (%).

In summary, this suggests that MxA, among other restriction factors, is an important determinant for the subtype-specific anti-IAV activity of IFN-α subtypes.

## Discussion

IFN-α subtypes are important signalling molecules for the innate immune response against viruses and other pathogens and were suggested to exhibit subtype and virus specific antiviral activities.

In this study, we compared the antiviral activities of all human IFN-α subtypes against seasonal H3N2 IAV in primary human lung tissue and provide experimental evidence that subtypes α16, α5 and α4 are up to 230-fold more potent to suppress H3N2 IAV replication than clinically licensed subtype α2 (Figure 4), which emphasizes their great potential as new anti-IAV treatments. So far, antiviral activities were only tested against a single seasonal H3N2 strain. Investigations using other seasonal strains, including H1N1, need to be performed to consolidate our findings on a broader scale. In addition, diverse drug resistant virus strains should be tested to verify their susceptibility to IFN-α treatments.

Our analysis of the innate immune response to H3N2 IAV infection in human lung tissue revealed that only five IFN-α subtypes (α1, α2, α8, α14 and α17) (Figure 3) were significantly upregulated after infection. Intriguingly, these subtypes exhibited only weak to intermediate antiviral activity against H3N2 in our analysis (Figure 4(A and C)) while the most potent subtypes α16, α5 and α4 were not induced. Therefore, our data suggest that exogenous application of the IFN-α subtypes with strong antiviral activities, such as α16, α5 and α4, could be a better option for antiviral treatment against IAV than the commonly used α2. Our results are in line with reports showing differential upregulation of IFN-α subtypes during infection with HIV [38,44], which reported strongest upregulation of α14, α2 and α10. This indicates that the pattern of induced subtypes could depend on the infecting virus. In contrast to our results, the authors of this study did not find a preference for induction of IFN-α subtypes with weak antiviral activity, as α14 was highly induced and exhibited high antiviral activity.

The molecular mechanisms underlying the selective upregulation of IFN-α subtypes during viral infections are unknown. It is speculated that virus infections trigger the activation of distinct signalling pathways, possibly by virus-specific PAMPs and engagement of different PRRs, which is responsible for the induction of virus-specific IFN-α subtype signatures. Alternatively, the upregulation of IFN-α subtypes could be determined by virus-specific antagonistic mechanisms, which selectively inhibit the expression of certain IFN-α subtypes. We cannot exclude that the observed pattern of IFN-α subtypes reflects the organ-specific response to the infection with H3N2 IAV in the human lower respiratory tract.

We investigated whether the increased antiviral activities of α16, α5 and α4 were linked to higher expression levels of antiviral ISG compared to weaker subtypes α1 and α2. Indeed, all three subtypes induced higher levels of IAV-specific restriction factors *MX1, DDX58, ISG15* and *EIF2AK2* (Figure 6(A)), but there was no evidence that one of these factors was of superior importance for subtype-specific antiviral activity against H3N2 IAV, as shown by the low correlation values (Figure 6(B)). Intriguingly, higher expression of virus-specific restriction factors by highly potent IFN-α subtypes was also observed for HIV [38]. In this report, α8 and α14, which were the strongest inhibitors of HIV infection, induced higher expression levels of the HIV-specific restriction factors *MX2*, *BST2* (Thetherin) and *APOBEC3G*. These observations suggest that the IFN-α family has evolved to provide a virus-tailored component of the host immune response against diverse virus infections.

MxA is a potent inhibitor of IAV replication and was strongly upregulated after treatment with α16, α5 and α4, suggesting that it is an important mediator of IFN-α antiviral activity. Nevertheless, we did not observe a linear correlation between the induction of its mRNA levels and the strength of the subtype-specific IAV inhibition. This led us to challenge the importance of MxA for the subtype-specific IAV restriction in A549 cells depleted for MxA. As expected, the inhibition of viral replication after IFN-α treatment was only partially abolished in the absence of MxA. However, we found that the antiviral activities of α16, α5 and α4 were differentially affected by the absence of MxA and that α5 demonstrated the strongest dependency on MxA (Figure 8(B) and Table 2). This suggests that the antiviral activity of IFN-α subtypes towards H3N2 IAV is not exclusively mediated by the action of MxA but most likely relies on the concerted actions of several ISGs. Furthermore, the differences in the dependency on MxA support the hypothesis that IFN-α subtypes induce subtype-specific ISG landscapes.

So far, it has remained elusive how IFN-α subtypes are able to induce individual biological activities despite binding to the same receptor. It was suggested that different binding affinities to the IFNAR receptor could affect downstream signalling and ISG induction [45]. Additionally, differences in the availability or distribution of the IFNAR chains 1 and 2 on the surface of specific cells types may also be important factors. Thus, studies focusing on the investigation of the biological functions of IFN-α subtypes will strongly benefit from primary tissue models. Due to these reasons, we analysed the antiviral potential of IFN-α subtypes against H3N2 IAV in human lung explants. To date, human lung tissue provides the unique possibility to study virus-host interactions of IAV in its natural target tissue [41]. The native alveolar 3D-architecture as well as the cellular diversity of the human lower respiratory tract (LRT) are preserved in this tissue and allow natural inter-cellular communications during viral infections. Most importantly, this model displays the natural patient heterogeneity and provides more realistic insights into the complex processes of the human immune response.

Results by us and others have shown that lung explants support the replication of diverse IAV strains as well as the induction of innate immune responses following viral infection or treatment with IFNs [41]. Especially for the analysis of IFNs and their therapeutic potential in humans, interspecies differences strongly recommend the use of primary human study models in order to provide clinically relevant results [46,47]. Limitation of *ex vivo* models is given by the absence of adaptive immune responses. Nevertheless, induction of pro-inflammatory cytokines and chemokines, such as IL-6, IL-8, MIP-1β, IP-10, IL-12p70 and IFNs, that are involved in bridging innate and adaptive immune responses by the recruitment and activation of immune cells, was readily observed in human lung tissue in this study (Figure 2).

In conclusion, our study provides the first comprehensive analysis of all human IFN-α subtypes against H3N2 IAV in primary human lung tissue. Our data demonstrate that α16, α5 and α4 possess superior antiviral activities against H3N2 IAV compared to licensed IFN-α2. In contrast to IFN-α2, subtypes α16, α5 and α4 were not induced upon infection with H3N2 in human lung tissue, but induced significant higher expression levels of IAV-specific restriction factors when exogenously applied to human lung tissue. Based on the results of our study, we strongly suggest the inclusion of these subtypes into future studies investigating IFN-α-based antiviral approaches against IAV.

## Supplementary Material

Supplemental Material

## References

[1] World Health Organization. Vaccines against influenza WHo position paper – November 2012. Wkly Epidemiol Rec. 2012;87:461–476. 23210147

[2] Iuliano AD, Roguski KM, Chang HH, et al. Estimates of global seasonal influenza-associated respiratory mortality: a modelling study. Lancet. 2018;391:1285–1300. doi: 10.1016/S0140-6736(17)33293-2 29248255 PMC5935243

[3] World Health Organization. Review of global influenza activity, October 2016– October 2017. Wkly Epidemiol Rec. 2017;92:761–780.29250946

[4] Soema PC, Kompier R, Amorij JP, et al. Current and next generation influenza vaccines: formulation and production strategies. Eur J Pharm Biopharm. 2015;94:251–263. doi: 10.1016/j.ejpb.2015.05.023 26047796

[5] World Health Organization. Recommended composition of influenza virus vaccines for use in the 2018–2019 northern hemisphere influenza season. Wkly Epidemiol Rec. 2018;93:133–152.29569429

[6] Hussain A, Ali S, Ahmed M, et al. The anti-vaccination movement: a regression in modern medicine. Cureus. 2018;10:e2919. 30186724 10.7759/cureus.2919 PMC6122668

[7] Frieden TR, Harold Jaffe DW, Director for Science James Stephens AW, et al. Antiviral agents for the treatment and chemoprophylaxis of influenza recommendations of the advisory committee on immunization practices (ACIP), centers for disease control and prevention. *MMWR* 2011; 6060. , [cited 2019 Feb 25]. Available from: http://www.cdc.gov/flu.

[8] Ison MG. Antiviral treatments. Clin Chest Med. 2017;38:139–153. doi: 10.1016/j.ccm.2016.11.008 28159156 PMC7131036

[9] Leneva IA, Burtseva EI, Yatsyshina SB, et al. Virus susceptibility and clinical effectiveness of anti-influenza drugs during the 2010–2011 influenza season in Russia. Int J Infect Dis. 2016;43:77–84. doi: 10.1016/j.ijid.2016.01.001 26775570

[10] Hayden FG, Sugaya N, Hirotsu N, et al. Baloxavir marboxil for uncomplicated influenza in adults and adolescents. N Engl J Med. 2018;379:913–923. doi: 10.1056/NEJMoa1716197 30184455

[11] Takashita E, Ejima M, Ogawa R, et al. Antiviral susceptibility of influenza viruses isolated from patients pre- and post-administration of favipiravir. Antiviral Res. 2016;132:170–177. doi: 10.1016/j.antiviral.2016.06.007 27321665

[12] Ludwig S. Targeting cell signalling pathways to fight the flu: towards a paradigm change in anti-influenza therapy. J Antimicrob Chemother. 2009;64:1–4. doi: 10.1093/jac/dkp161 19420020

[13] Hoofnagle JH, Seeff LB. Peginterferon and ribavirin for chronic hepatitis C. N Engl J Med. 2006;355:2444–2451. doi: 10.1056/NEJMct061675 17151366

[14] Díaz MO, Pomykala HM, Bohlander SK, et al. Structure of the human type-I interferon gene cluster determined from a YAC clone contig. Genomics. 1994;22:540–552. doi: 10.1006/geno.1994.1427 8001965

[15] Heil F, Hemmi H, Hochrein H, et al. Species-Specific recognition of single-Stranded RNA via Toll-like receptor 7 and 8. Proc Natl Acad Sci USA. 2013;303:1526–1529. 10.1126/science.1093620 14976262

[16] Kawai T, Akira S. The role of pattern-recognition receptors in innate immunity: update on toll-like receptors. Nat Immunol. 2010;11:373–384. doi: 10.1038/ni.1863 20404851

[17] Kim N, Now H, Nguyen NTH, et al. Multilayered regulations of RIG-I in the anti-viral signaling pathway. J Microbiol. 2016;54:583–587. doi: 10.1007/s12275-016-6322-2 27572506

[18] Darnell JE, Kerr LM, Stark GR. Jak-STAT pathways and transcriptional activation in response to IFNs and other extracellular signaling proteins downloaded from. 1994. [cited 2019 Mar 14]. Available from: http://science.sciencemag.org/. 10.1126/science.8197455 8197455

[19] Song J, Li S, Zhou Y, et al. Different antiviral effects of IFNα subtypes in a mouse model of HBV infection. Sci Rep. 2017;7:334. doi: 10.1038/s41598-017-00469-1 28336921 PMC5428457

[20] Matzinger SR, Carroll TD, Fritts L, et al. Exogenous ifn-alpha administration reduces influenza a virus replication in the lower respiratory tract of rhesus macaques. PLoS One. 2011;6:e29255, doi:10.1371/journal.pone.0029255. 22220209 PMC3248419

[21] Matzinger SR, Ma Z-M, Miller CJ, et al. Myxovirus resistance gene a (MxA) expression suppresses influenza a virus replication in alpha interferon-treated primate cells. J Virol. 2013;87:1150–1158. doi: 10.1128/JVI.02271-12 23152507 PMC3554078

[22] Arimori Y, Nakamura R, Yamada H, et al. Type I interferon limits influenza virus-induced acute lung injury by regulation of excessive inflammation in mice. Antiviral Res. 2013;99:230–237. doi: 10.1016/j.antiviral.2013.05.007 23721943

[23] Kugel D, Kochs G, Obojes K, et al. Intranasal administration of alpha interferon reduces seasonal influenza a virus morbidity in ferrets. J Virol. 2009;83:3843–3851. doi: 10.1128/JVI.02453-08 19193792 PMC2663257

[24] Hayden FG, Gwaltney JM. Intranasal interferon- 2, treatment of experimental Rhinoviral Colds. J Infect Dis. 1984;150:174–180. doi: 10.1093/infdis/150.2.174 6381610

[25] Treanor JJ, Betts RF, Erb SM, et al. Intranasally administered interferon as prophylaxis against experimentally induced influenza a virus infection in humans. J Infect Dis. 1987;156:379–383. doi: 10.1093/infdis/156.2.379 3598236

[26] Sarno TC, Greenberg SB, Couch RB, et al. Efficacy and tolerance of intranasally applied recombinant leukocyte a interferon in normal volunteers. J Infect Dis. 1983;148:535–542. doi: 10.1093/infdis/148.3.535 6619578

[27] Gao LL, Yu S, Chen Q, et al. A randomized controlled trial of low-dose recombinant human interferons α-2b nasal spray to prevent acute viral respiratory infections in military recruits. Vaccine. 2010;28:4445–4451. doi: 10.1016/j.vaccine.2010.03.062 20394720 PMC7115383

[28] Bennett AL, Smith DW, Cummins MJ, et al. Low-dose oral interferon alpha as prophylaxis against viral respiratory illness: a double-blind, parallel controlled trial during an influenza pandemic year. Influenza Other Respi Viruses. 2013;7:854–862. doi: 10.1111/irv.12094 PMC5781220 23398960

[29] James CM, Abdad MY, Mansfield JP, et al. Differential activities of alpha/beta IFN subtypes against influenza virus in vivo and enhancement of specific immune responses in DNA vaccinated mice expressing haemagglutinin and nucleoprotein. Vaccine. 2007;25:1856–1867. doi: 10.1016/j.vaccine.2006.10.038 17240000

[30] Scagnolari C, Trombetti S, Soldà A, et al. Pandemic 2009 H1N1 influenza virus is resistant to the antiviral activity of several interferon alpha subtypes. J Interf Cytokine Res. 2011;31:475–479. doi: 10.1089/jir.2010.0125 21235413

[31] Moll HP, Maier T, Zommer A, et al. The differential activity of interferon-α subtypes is consistent among distinct target genes and cell types. Cytokine. 2011;53:52–59. doi: 10.1016/j.cyto.2010.09.006 20943413 PMC3020287

[32] Dobin A, Gingeras TR. Mapping RNA-seq reads with STAR. Curr Protoc Bioinforma. 2015;51:11.14.1–19. doi: 10.1002/0471250953.bi1114s51 PMC4631051 26334920

[33] Liao Y, Smyth GK, Shi W. The R package rsubread is easier, faster, cheaper and better for alignment and quantification of RNA sequencing reads. Nucleic Acids Res. 2019;47:e47. doi: 10.1093/nar/gkz114 30783653 PMC6486549

[34] R Core Team. R: A Language and Environment for Statistical Computing. R Foundation for Statistical Computing. 2013. Available from: http://www.r-project.org/.

[35] Love MI, Huber W, Anders S. Moderated estimation of fold change and dispersion for RNA-seq data with DESeq2. Genome Biol. 2014;15:550. doi: 10.1186/s13059-014-0550-8 25516281 PMC4302049

[36] Smyth GK. Linear models and empirical bayes methods for assessing differential expression in microarray experiments. Stat Appl Genet Mol Biol. 2004;3:1–25. doi: 10.2202/1544-6115.1027 16646809

[37] Benjamini Y, Hochberg Y. Controlling the false discovery rate: a practical and powerful apporach to multiple testing. J R Stat Soc. 1995;57:289–300.

[38] Harper MS, Guo K, Gibbert K, et al. Interferon-α subtypes in an ex vivo model of acute HIV-1 infection: expression, potency and effector mechanisms. PLoS Pathog. 2015;11:e1005254. doi: 10.1371/journal.ppat.1005254 26529416 PMC4631339

[39] Zimmermann A, Trilling M, Wagner M, et al. A cytomegaloviral protein reveals a dual role for STAT2 in IFN-γ signaling and antiviral responses. J Exp Med. 2005;201:1543–1553. doi: 10.1084/jem.20041401 15883169 PMC2212917

[40] Shalem O, Sanjana NE, Hartenian E, et al. Genome-scale CRISPR-Cas9 knockout screening in human cells. Science. 2014;343:84–87. doi: 10.1126/science.1247005 24336571 PMC4089965

[41] Weinheimer VK, Becher A, Tönnies M, et al. Influenza a viruses target type II pneumocytes in the human lung. J Infect Dis. 2012;206:1685–1694. doi: 10.1093/infdis/jis455 22829640 PMC7107318

[42] Sato S, Li K, Kameyama T, et al. The RNA Sensor RIG-I Dually functions as an innate Sensor and direct antiviral factor for hepatitis B virus. Immunity. 2015;42:123–132. doi: 10.1016/j.immuni.2014.12.016 25557055

[43] Weber M, Sediri H, Felgenhauer U, et al. Influenza virus adaptation PB2-627K modulates nucleocapsid inhibition by the pathogen sensor RIG-I. Cell Host Microbe. 2015;17:309–319. doi: 10.1016/j.chom.2015.01.005 25704008 PMC4359673

[44] Li Y, Sun B, Esser S, et al. Expression pattern of individual *IFNA* subtypes in chronic HIV infection. J Interf Cytokine Res. 2017;37:541–549. doi: 10.1089/jir.2017.0076 29252127

[45] Lavoie TB, Kalie E, Crisafulli-Cabatu S, et al. Binding and activity of all human alpha interferon subtypes. Cytokine. 2011;56:282–289. doi: 10.1016/j.cyto.2011.07.019 21856167

[46] Zscheppang K, Berg J, Hedtrich S, et al. Human pulmonary 3D models For translational research. Biotechnol J. 2018;13:1700341. doi: 10.1002/biot.201700341 28865134 PMC7161817

[47] Hocke AC, Suttorp N, Hippenstiel S. Human lung ex vivo infection models. Cell Tissue Res. 2017;367:511–524. doi: 10.1007/s00441-016-2546-z 27999962 PMC7087833

