# Supplementary material for: Antiviral potential of human IFN-α subtypes against influenza A H3N2 infection in human lung explants reveals subtype-specific activities
Source: Emerg Microbes Infect. 2019 Dec 11;8(1):1763–76. doi: 10.1080/22221751.2019.1698271 (PMC6913622; doi:10.1080/22221751.2019.1698271)
Supplement: Supplemental Material [file TEMI_A_1698271_SM6317.zip › suppl_data/Supplementary_Table_1_final.docx]

**Supplementary Table 1 - Primer sequences used for RT-qPCR**

**qPCR Primer**  **Sequence**

huGAPDH fwd 5’- gcaaattccatggcaccgt -3’

huGAPDH rev 5’- gccccacttgatttggagg -3’

huMxA fwd 5’- gtttccgaagtggacatcgca -3’

huMxA rev 5’- gaagggcaactcctgacagt -3’

huRIG-I fwd 5’- CCTACCTACATCCTGAGCTACAT -3’

huRIG-I rev 5’- TCTAGGGCATCCAAAAAGCCA -3’

huOAS1 fwd 5’- GATCTCAGAAATACCCCAGCCA -3’

huOAS1 rev 5’- AGCTACCTCGGAAGCACCTT -3’

huISG15 fwd 5’- TCCTGGTGAGGAATAACAAGGG -3’

huISG15 rev 5’- GTCAGCCAGAACAGGTCGTC -3’

huIFNγ fwd 5’- AACCGAGATGACTTCGAAAAGCTG -3’

huIFNγ rev 5’- TGTTTAGCTGCTGGCGACAG -3’

huIL6 fwd 5’- aacctgaaccttccaaagatgg-3’

huIL6 rev 5’- tctggcttgttcctcactagt-3’

huPKR fwd 5’- ACTTCTGCCTCTTGGGTTCA -3’

huPKR rev 5’- GGGGTGGTTTTTCCATCTTT -3’

huMCP1 fwd 5’- TGCCCTCCATCATGAAAGTC -3’

huMCP1 rev 5’- TTGCATCTGGCTGAGCGAG -3’

huMIP1β fwd 5’- CTGTGCTGATCCCAGTGAATC -3’

huMIP1β rev 5’- TCAGTTCAGTTCCAGGTCATACA -3’
