# Supplementary figures and images for: Antiviral potential of human IFN-α subtypes against influenza A H3N2 infection in human lung explants reveals subtype-specific activities
Source: Emerg Microbes Infect. 2019 Dec 11;8(1):1763–76. doi: 10.1080/22221751.2019.1698271 (PMC6913622; doi:10.1080/22221751.2019.1698271)

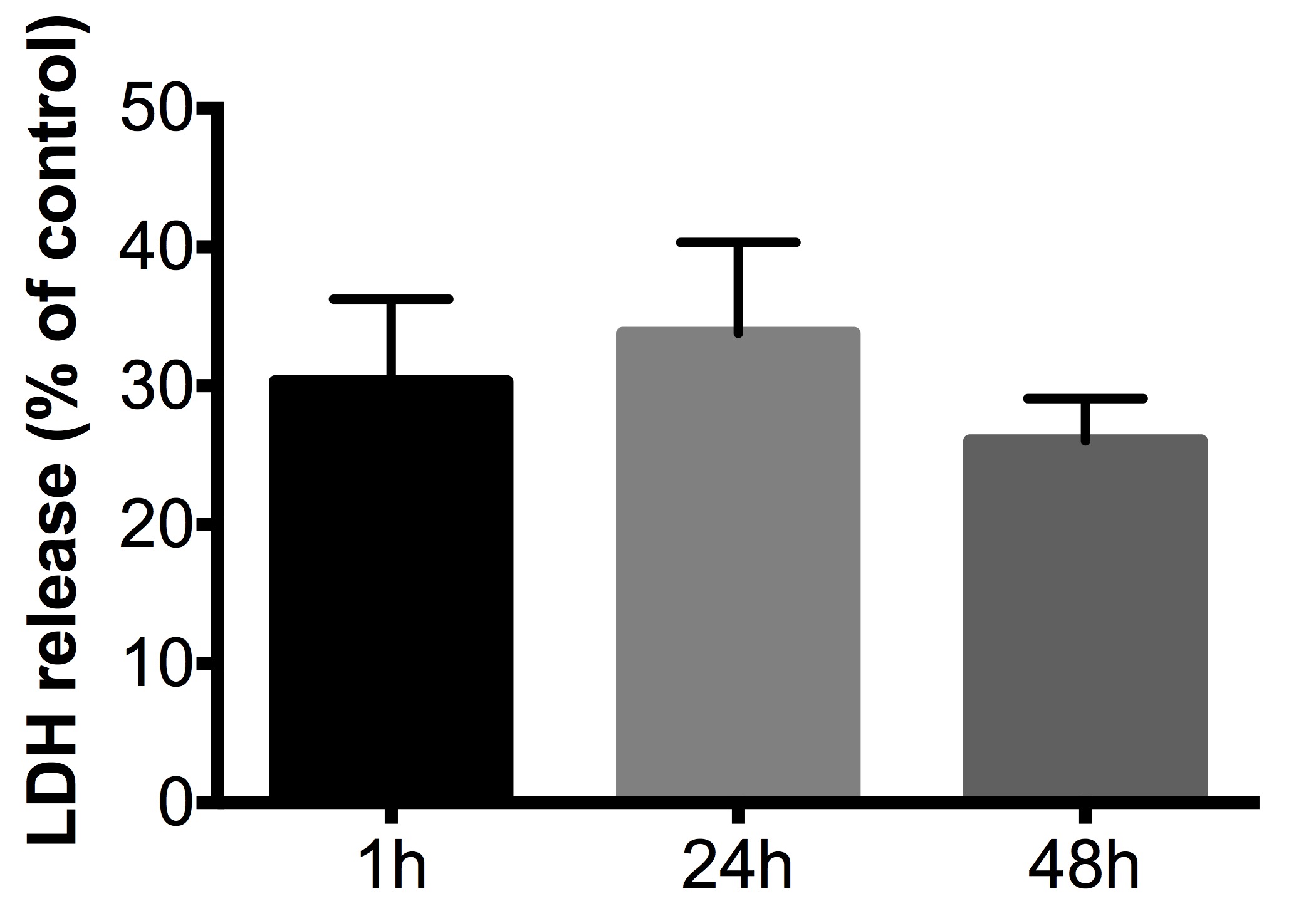

Supplement: Supplemental Material [file TEMI_A_1698271_SM6317.zip › suppl_data/supplementary figure 1.jpg]

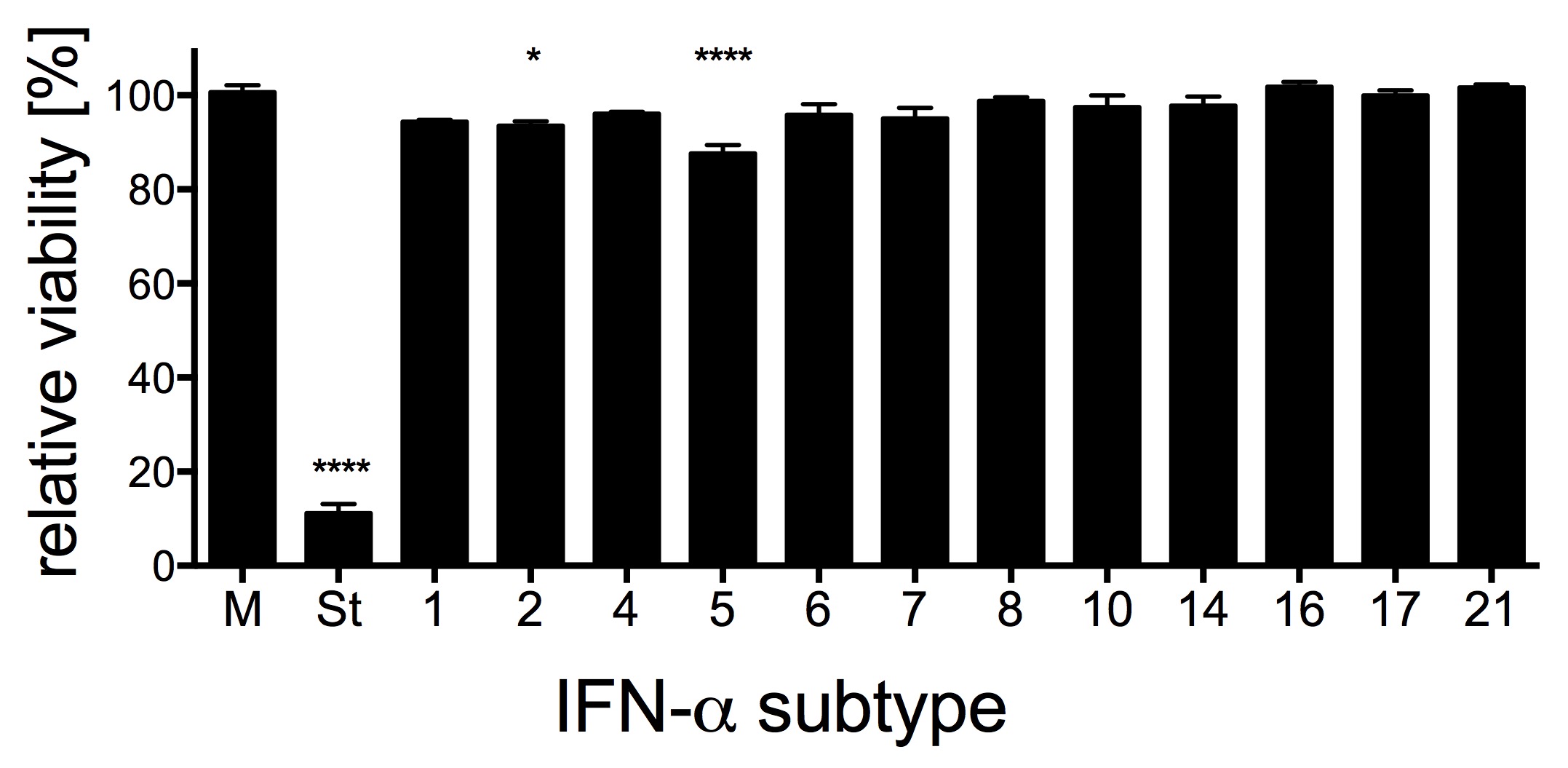

Supplement: Supplemental Material [file TEMI_A_1698271_SM6317.zip › suppl_data/supplementary figure 2.jpg]

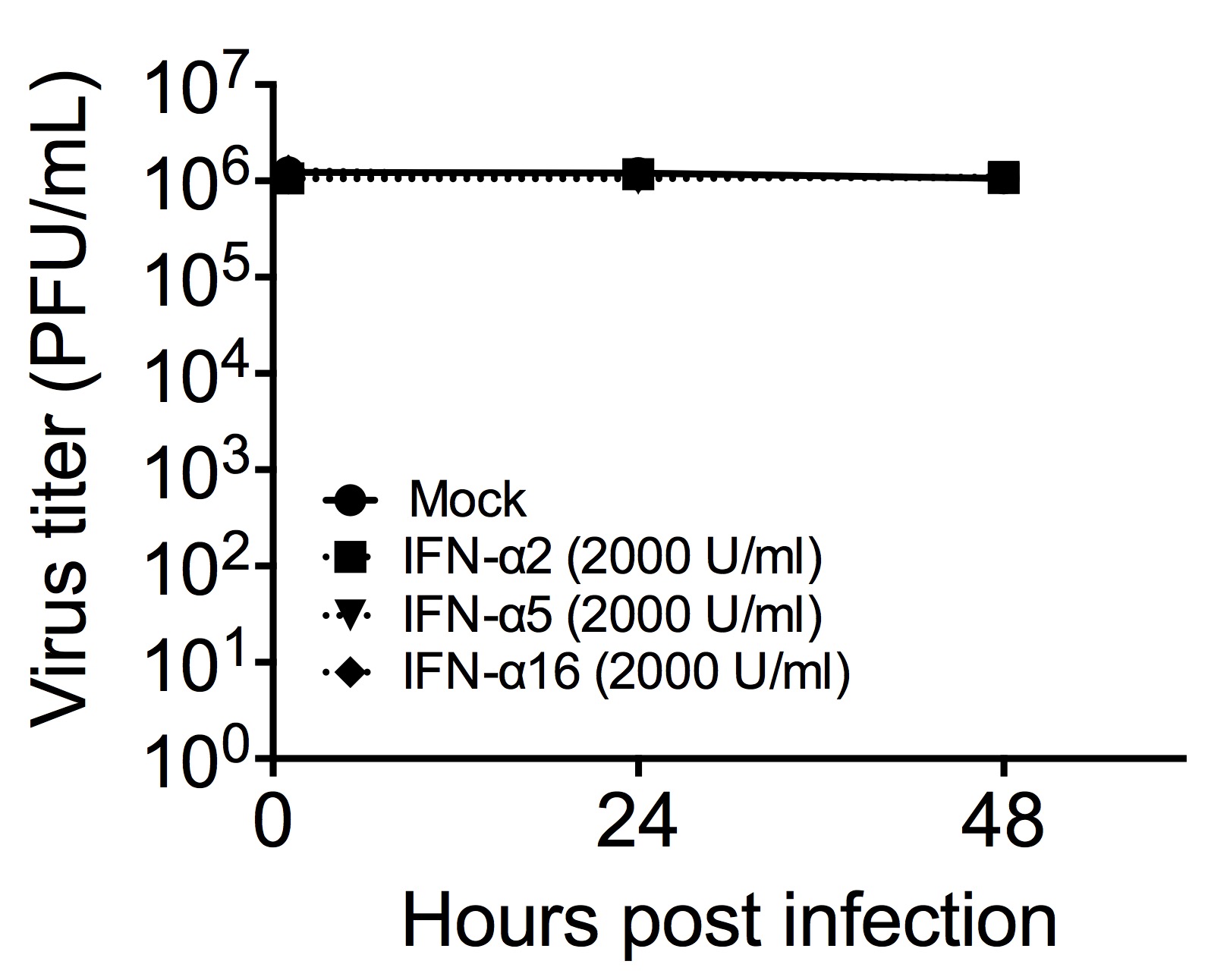

Supplement: Supplemental Material [file TEMI_A_1698271_SM6317.zip › suppl_data/supplementary figure 3.jpg]

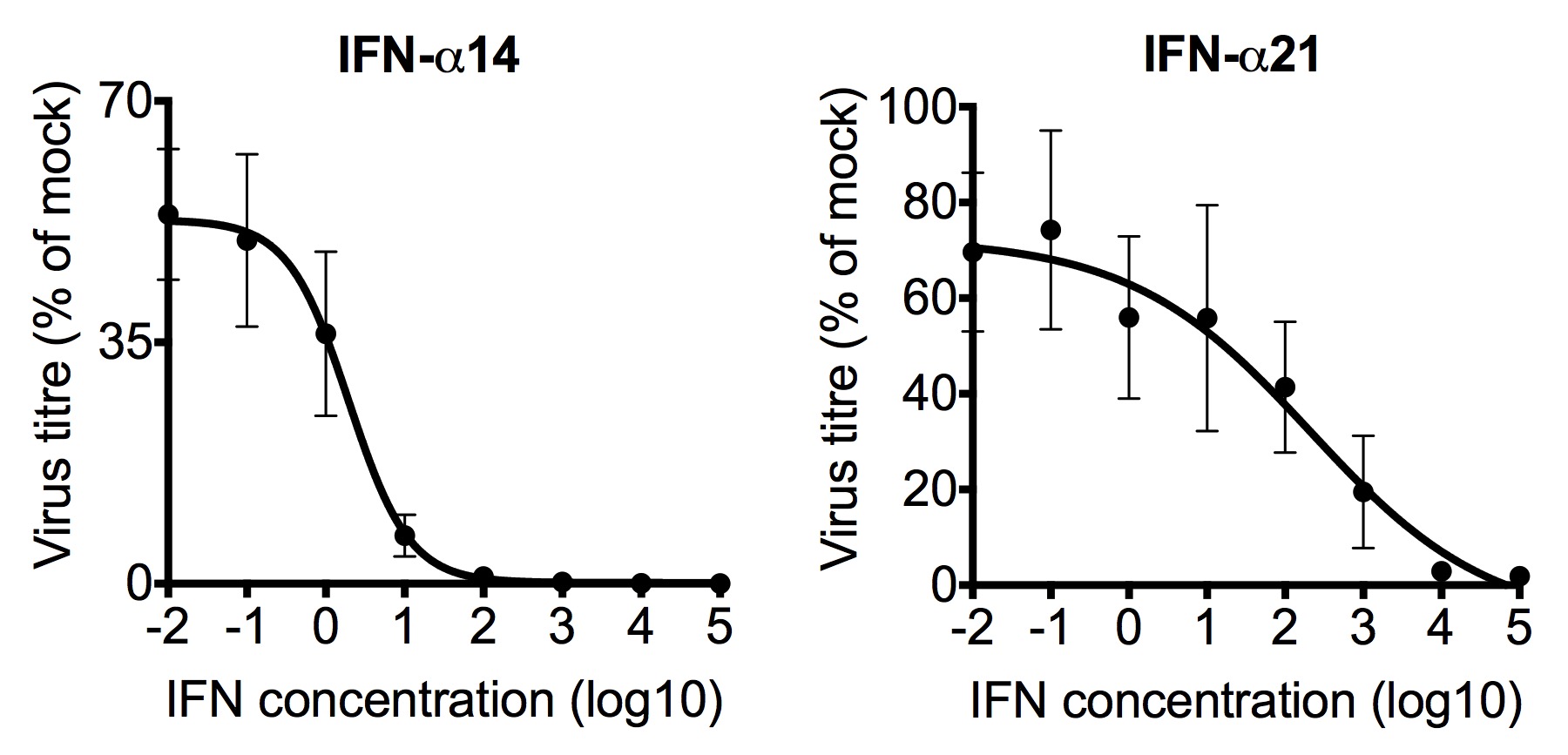

Supplement: Supplemental Material [file TEMI_A_1698271_SM6317.zip › suppl_data/Supplementary Figure 4.jpg]
